# Girls-Boys: An Investigation of Gender Differences in the Behavioral and Neural Mechanisms of Trust and Reciprocity in Adolescence

**DOI:** 10.3389/fnhum.2019.00257

**Published:** 2019-08-02

**Authors:** Imke L. J. Lemmers-Jansen, Anne-Kathrin J. Fett, Sukhi S. Shergill, Marlieke T. R. van Kesteren, Lydia Krabbendam

**Affiliations:** ^1^Department of Clinical, Neuro and Developmental Psychology, Faculty of Behavioral and Movement Sciences, Institute for Brain and Behavior, Vrije Universiteit Amsterdam, Amsterdam, Netherlands; ^2^Department of Psychology, City, University of London, London, United Kingdom; ^3^Department of Psychosis Studies, King’s College London, Institute of Psychiatry, Psychology and Neuroscience, London, United Kingdom; ^4^Department of Education Sciences, Faculty of Behavioral and Movement Sciences, Institute for Brain and Behavior, Vrije Universiteit Amsterdam, Amsterdam, Netherlands

**Keywords:** trust, reciprocity, gender, development, mentalizing, reward, fMRI

## Abstract

**Background:**

Trust and reciprocity toward others have often been found to increase from childhood to adulthood. Gender differences in these social behaviors have been reported in adults. While adolescence is a key-period of change in social behavior, gender differences in trust and reciprocity during this developmental stage have rarely been investigated.

**Methods:**

Here we investigate age-related gender differences in trust and reciprocity (*n* = 100, 51 female) and associated neural mechanisms (*n* = 44, 20 female) in adolescents between 13 and 19 years of age. Participants played two multi-round trust games with a pre-programmed cooperative and an unfair partner. Forty-four of 100 participants completed the trust game while undergoing functional brain imaging.

**Results:**

Participants’ investments were greater toward a cooperative than unfair game partner (*p* < 0.01), showing sensitivity to the degree of trustworthiness. There were no gender or age or related differences in baseline trust. In repeated cooperative interactions no gender differences were found, but younger adolescents showed slightly steeper increase of investments than older adolescents. In unfair interactions, younger males reacted with stronger decrease of investments than older males. Region of interest analysis of brain areas associated with in mentalizing, reward learning, conflict processing, and cognitive control revealed gender-by-age interactions on trusting behavior in the temporo-parietal junction (TPJ) and the caudate, showing stronger influence of age in males than in females during cooperation, and the reverse in unfair interactions. Additionally, main effects of gender were found in the TPJ, with higher activation in males, and in the caudate, with females showing greater activation.

**Conclusion:**

In first interactions and during repeated cooperative interactions, adolescent males and females showed similar trusting behavior. Younger males showed stronger responses to unfairness by others. Gender-by-age interactions in specific ROIs suggest differential development in mentalizing and reward related cognitive processes. In conjunction with previous research, our findings suggest the presence of subtle gender and age-related changes in trust and cooperation that are only detectable using larger age windows.

## Introduction

Adolescence is a period of marked changes in social orientation, shifting from a family focus toward peer relations (Steinberg and Sheffield Morris, 2001; Brown, B.B., 2004; Nelson et al., 2005; Crone and Dahl, 2012). This development is supported by ongoing maturation of social (cognitive) skills. A crucial skill is the ability to trust and recognize trustworthiness in others. Trust is essential to initiate, establish, and maintain social relationships, by making relationships more cooperative and satisfactory, and strengthening norms that favor cooperation and/or increase group outcome (Balliet and Van Lange, 2013). Trust is associated with expectations, predictability, and confidence in others’ behavior, with an emphasis on the benevolent motives of others in situations that involve a conflict between own interests and the interest of others (Balliet and Van Lange, 2013). The shift from a family focus toward peer relations in adolescence also encompasses a change from unconditional trust in close relatives to learning to trust people outside the family circle. Learning to trust others occurs in a process of repeated interactions that make it possible to build a mental model of the behavior of the other person. To initiate positive, cooperative interactions, trust in the positive reciprocity of the other is essential. For the maintenance of these interactions and for building social relationships, reciprocation of the initial trust is necessary (van den Bos et al., 2010). Initial distrust may be overcome by positive reciprocity, indicating that trust may grow in response to reciprocal behavior. Motivations to trust may vary (e.g., intrinsic, altruistic vs. extrinsic strategic), and both cognitive and affective processes play a role (Evans and Krueger, 2011; Balliet and Van Lange, 2013; Cutler and Campbell-Meiklejohn, 2019). In this study, trust is operationalized by means of the height of investments in the trust game (Berg et al., 1995).

In the trust game participants share a part of a given amount of money with an unknown person. The amount is tripled and the second person may return a certain amount to the investor, or keep it all. Trust in this paradigm is defined and operationalized as sending an endowment, so that the trustee can choose to honor trust, or not (Berg et al., 1995). The trust game allows to investigate baseline trust (i.e., the first investment given to an unknown person), as an index of a person’s general inclination to trust. Additionally, in a multi-round trust game a context is created, in which trust can emerge as the outcome of a sustained social relationship (Cochard et al., 2004). In repeated interactions, the investor responds to the social feedback, adjusting the levels of trust accordingly (Tzieropoulos, 2013). Investigating trust in an experimental manner involves making commitments for real amounts of money, therefore resembling daily life situations more than questionnaires (Cochard et al., 2004). Experiments also allow for the systematic manipulation of context (response patterns of the trustee), yielding comparable data due to identical settings for all participants and added measures, such as neural data during task performance, acquired with functional Magnetic Resonance Imaging (fMRI).

Previous research yielded important insights into the development of trust and social mechanisms, such as reciprocity and cooperation (Eisenberg et al., 2002, 2005; Cochard et al., 2004; Steinberg, 2005; van den Bos et al., 2010, 2011, 2012; Smith et al., 2013; Fett et al., 2014b), and into gender differences in trust (Croson and Buchan, 1999; Balliet et al., 2011; Chaudhuri and Sbai, 2011; Chaudhuri et al., 2013; Van den Akker, 2018). People become more inclined to trust and to establish cooperation from childhood and early adolescence until middle adulthood (Sutter and Kocher, 2007; van den Bos et al., 2010, 2012; Evans et al., 2013; Fett et al., 2014a). Sutter and Kocher (2007) found that trust increases linearly (age 8–60^+^) until 22 years of age, showing stability in adulthood and a slight decrease thereafter; Van den Bos and colleagues reported increasing trust from childhood to mid-adolescence and a slight decrease toward early adulthood (age 9–25) (van den Bos et al., 2010), as well as increased first investments and enhanced learning over trials with age (van den Bos et al., 2012). In very young children (age 4–5 and 9–10), trust was found to increase by 6-fold between kindergarten and elementary school, even when controlling for altruism (Evans et al., 2013). In contrast to the aforementioned studies, where different age groups were compared, research within a smaller age-range has shown a decrease of trust in adolescents aged 14–16.5 (Derks et al., 2014), or stable levels of trust between 12 and 18 years (van de Groep et al., 2018). These findings suggest that trust may develop until the early twenties, thereafter stabilizing or slightly decreasing, but the findings are contradictory about the exact time window of development.

Trust not only differs between developmental stages, but also between genders. During repeated interactions, males have been found to display more trust than females (Croson and Gneezy, 2009; Balliet et al., 2011). However, in negative, unfair interactions where trust is not reciprocated, females are more likely to stay trusting and to restore trust (Haselhuhn et al., 2015). Similarly, trust in unknown others differs between the genders, both in adolescents (Derks et al., 2014; van de Groep et al., 2018), and in adults (Buchan et al., 2008; Croson and Gneezy, 2009; Van den Akker, 2018), showing that men are more trusting than women. Only few studies have investigated gender differences and development of trust experimentally. In young children, age 4–5 girls trusted more often than boys, but a few years later (age 9–10), the reverse was found, resembling adult data (Evans et al., 2013). In a previous study, we have shown that during late-adolescence and early adulthood, males displayed higher baseline trust than females, and males reduced their trust more drastically with increasing age than females in interactions in which trust is not reciprocated (Lemmers-Jansen et al., 2017).

At the neural level, the motivation to cooperate is proposed to be modulated by the cognitive control system (centered on the dlPFC), regions of the social brain including the temporo-parietal junction (TPJ), the medial prefrontal cortex (mPFC), and the amygdala (Declerck et al., 2013), the anterior insula (Bellucci et al., 2016; Cutler and Campbell-Meiklejohn, 2019), and reward predicting areas, such as the caudate (Rilling et al., 2002; King-Casas et al., 2005; Tabibnia and Lieberman, 2007; Krill and Platek, 2012; Bellucci et al., 2016). Gender differences in neural activation during the trust game have shown increased activation of the TPJ in males compared to females, and increased activation of the caudate in females in a sample of late-adolescents and young adults (Lemmers-Jansen et al., 2017). Investigating trust in e-Bay offers in adults (30–35 years), females activated more striatal, whereas males activated more prefrontal areas (Riedl et al., 2010). Many of these regions are still developing during adolescence (Nelson et al., 2005; Blakemore, 2012; Crone and Dahl, 2012; Harenski et al., 2012). In the trust game, age-related increases of activation were found in the TPJ, posterior cingulate, right dorsolateral prefrontal cortex (dlPFC), right caudate, and precuneus (Fett et al., 2014b; Lemmers-Jansen et al., 2017). Age-related reductions in activation were also reported in the orbitofrontal cortex and caudate during interactions with a trustworthy, cooperative partner (Fett et al., 2014b), and in the anterior medial prefrontal cortex (amPFC) (van den Bos et al., 2011). In sum, previous findings suggest that differential neural activation patterns in brain areas involved in mentalizing, reward learning and cognitive control are associated with gender differences and age-related changes in trust and reciprocity toward others.

### The Current Study

This study set out to investigate gender differences in the development and the underlying neural mechanisms of trust and reciprocity in adolescents (age 13–19). Participants played two repeated trust games, one with a cooperative partner, always returning the invested amount or more, and one with an unfair partner, who always returned less than invested. In our older adolescent-early adult sample, gender differences were present in baseline trust and males reacted with a steeper decline in investment to unfair treatment by the other than females. This effect became more pronounced with age (Lemmers-Jansen et al., 2017). However, overall, we found relatively stable patterns of trust, with neural activation that did not change with age (e.g., suggesting maturity). Possibly, changes in trust occur earlier in development. In an attempt to pinpoint the possible time window, the current study extends findings of our previous study to a younger sample of adolescents, who are in the middle of this process of social reorientation. Due to differential developmental speed, the development of trust and reciprocity may differ between boys and girls (Lenroot and Giedd, 2010; Blakemore, 2012; Crone and Dahl, 2012). Furthermore, social demands may differ between boys and girls, resulting in differential socialization processes, which lead to increasing gender differences in trust over time (Rose and Rudolph, 2006). In the current study we investigate differences in development of social behavior over repeated social interactions in an experimental setting, using a neuroeconomic trust game. Analogous to our previous study, we used two multi-round trust games, one with a pre-programmed cooperative and one with an unfair partner. Participants played the role of the investor and could make continuous investments. We investigated gender differences in baseline trust (i.e., first investments) and in the modulation of trust in response to reciprocated trust (i.e., cooperation) and in interactions where trust was not reciprocated (i.e., unfairness). Based on the previously discussed literature in adults and older adolescents, we hypothesized gender differences in baseline trust, with higher trust in males than in females. Additionally, we explored the association between age and first investment (i.e., baseline trust). Over a larger age range increases of baseline trust have been reported (Fett et al., 2014a), however, this was not found in adolescent samples (Derks et al., 2014; van de Groep et al., 2018). Furthermore, based on the literature and our previous study, we hypothesized that males and females would show similar investments during cooperative interactions, but that males would show more reduction of investments during unfair interactions than females. In addition, we expected that with age, trust would increase during cooperative interactions, and decrease during unfair interactions, and that gender differences would become more pronounced. At the neural level we tested gender differences and associations with age in nine predefined regions of interest (ROI), associated with mentalizing, reward, cognitive control, and conflict processing. Finally, we explored in the ROIs whether gender and age effects differed between cooperative and unfair interactions.

## Materials and Methods

### Participants

Hundred healthy, right-handed adolescents, 51 female and 49 male, aged 13–19 (mean age = 16.5; *SD* = 1.57) participated in the behavioral part of this study. A subset of 24 males and 20 females also participated in fMRI. Part of the larger sample was previously described as the healthy comparison group for an early psychosis sample (Fett et al., 2016) and data of the males who took part in fMRI has previously been reported in a study that examined age effects in trust from adolescence to late adulthood (Fett et al., 2014a). For participant characteristics of this sub-sample, please see the Supplementary Material (Table S1). Participants were recruited at local schools in London, via colleagues and recruitment circulars at the Institute of Psychiatry, Psychology and Neuroscience. All participants had a good command of the English language. Participants had no history of neurological disorder, no psychiatric diagnosis, or psychotropic medication. Written informed consent was obtained from all participants and when under the age of 16 also from their parents/guardians. This study was approved by the research ethics committee London-Surrey Borders (10/H0806/38).

### Measures

#### WASI Vocabulary Scale

The vocabulary subtest of the Wechsler Abbreviated Scale of Intelligence (WASI) was used as indicator of general cognitive ability [13–18 years (Wechsler, 1999)], to investigate for possible confounding. T-scores were scaled for age.

#### Trust Game

**FIGURE 1:**
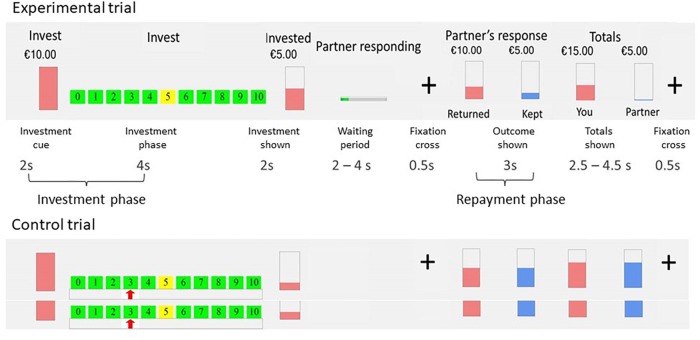
Graphical overview of the trust game. *Note:* Top row represents the visual stimuli in the game trials; middle row are the separate phases including durations of the trust game; bottom row represents the visual stimuli in the control trials. Taken with permission from Lemmers-Jansen et al. (2017).

Participants played the role of investor in two multi-round trust games. They were told that their two anonymous counterparts, the trustees, were connected to them via the Internet. In reality, they played against a computer, with two algorithms programmed to respond always in a cooperative and always in an unfair way. The algorithm was programmed in a probabilistic way: In the cooperative condition, with each increase in trust from the investor, the chance of a repayment of 200% increased with 10%. In the unfair condition, increases in trust from the investor increased the chance of a repayment of 50% (Gromann et al., 2013; Fett et al., 2014a, 2016). The two games were presented in counterbalanced order. Each game consisted of 20 experimental and 20 control trials. At the beginning of each experimental trial, participants started with $10. Any amount between $0 and $10 could be invested. The invested money was tripled and the trustee (i.e., computer) then made a repayment. Control trials were included as baseline condition for the fMRI analysis. The design and duration of the control trials were equal to the experimental trials, but without the element of investment. In the control trials participants had to move the cursor to a number between 0 and 10, which was indicated by a red arrow. Every trial started with an investment cue (2 s), followed by the investment period where participants made their choice (4 s, regardless of reaction times); the invested amount was shown (2 s), followed by a waiting period (jittered, 2–4 s), and a fixation cross (500 ms). Finally, the returned amount (3 s) and the final totals of both players (jittered, 2.5–4.5 s) were displayed, followed by a fixation cross (500 ms). Every trial lasted 18.5 s in total. For a graphical representation of the set-up of the trust game, see Figure 1. After the trust game, participants completed a short questionnaire that asked if at some point they had doubts that their counterpart was a real person (outcome represented in Table 1). About one third of the participants reported doubts. Therefore we report sensitivity analyses, comparing results of the participants with and without doubts. Additionally, all analyses were run including only participants without doubts that the trustee was real.

**Table 1:** Participant characteristics, trust game behavior and beliefs.

	Male *N* = 49	Female *N* = 51	Statistics		Overall *N* = 100
Measures	Mean (*SD*)	Mean (*SD*)	*Beta*	*p*	Mean (*SD*)
Age	16.35 (1.65)	16.58 (1.5)	0.08	0.45	16.47 (1.57)
WASI t score	55.31 (11.89)	49.55 (9.25)^∗^	0.26	0.008	52.37 (11.02)
First investment, baseline trust	6.29 (2.10)	5.61 (2.30)	0.12	0.24	5.94 (2.22)
Mean investment Cooperative partner	6.84 (2.89)	6.33 (2.76)	0.02	0.35	6.58 (2.83)
Mean investment Unfair partner	3.73 (3.25)	4.08 (2.88)	–0.04	0.13	3.91 (3.07)
	
	***N* (%)**	***N* (%)**	**χ^2^**	***p***	***N* (%)**
	
**After trust game questionnaire**^#^					
Manipulation doubt?	15 (32%)	13 (33%)	0.02	0.89	28 (32%)
Strategy:			2.52	0.64	
-responding to partner	21 (47%)	13 (31%)			34 (40%)
- maximize profit	9 (20%)	8 (20%)			17 (20%)
- no strategy	11 (24%)	15 (37%)			26 (30%)
- other	4 (9%)	5 (12%)			9 (10%)


*^∗^Significant difference at *p* < 0.01. ^#^data of nine participants missing in the manipulation questionnaire, and 14 missing for the strategy questions. *Note:* WASI vocabulary = Wechsler Abbreviated Scale of Intelligence, vocabulary subscale, scaled for age. After the trust game participants were asked if at any time they had doubts whether their counterpart was real. If they responded personalizing (saying “he”), it was coded as believing the counterpart was real. If in two conditions they reported probabilistic answers, predictable or unreal, then it was coded as having doubts. Then participants were asked about the strategy used for investment. If the answer included the behavior of the counterpart, it was coded as “responding to partner”; if the answer contained “great,” “maximum,” “profit,” or “more than the other” it was coded as “maximize profit”; some participants answered the did not use a strategy; and other comprises “random,” “gambling,” or always trying the same amount.*

### Procedure

After signing the consent form, participants were assessed with the WASI Vocabulary subtest. Other measures were administered, which are unrelated to the current topic. Before scanning participants completed 10 trust game practice rounds on a laptop. Participants were told that they were connected with their game partners via the Internet and that they would receive the earnings from one randomly selected round of the trust game. During scanning, two different runs of the trust game were administered, one with a cooperative and one with an unfair interaction partner, and structural scans were acquired. The complete scanning session lasted approximately one hour. After scanning the participants answered a short questionnaire, which examined their individual perceptions of the trust game and their game partners. Participants were given a fixed payment for participation, and for fairness reasons, all participants received $5 extra, as earnings from the trust game.

### Data Analysis

#### Analyses of Behavioral Data

We analyzed the behavioral data using StataSE LAB 14 (StataCorp, 2015). We analyzed the effect of the condition on the amounts of the investments to check if the participants responded to the differences in response patterns of their interaction partners, with the investment as the dependent variable, using multilevel random regression analyses (XTREG), to account for multiple observations [investments (level 1); within participants (level 2)]. To test our hypotheses regarding changes of trust, we used the same multilevel regression analyses, including gender, age, and trial number, and their interaction as predictors. Trial number indicates the changes over time during the game, the development of trust in response to social feedback. The WASI score was added as covariate, to control for possible confounding of verbal cognitive ability. Analyses were run separately for the cooperative and unfair condition. Additionally, the effects of gender and age on first investment (e.g., baseline trust) were investigated. Results were considered significant when *p* < 0.05.

#### fMRI Image Acquisition and Analyses

Imaging data were acquired using a 3 Tesla GE Signa Neuro-optimized MR System. A quadrature birdcage head coil was used for radio frequency transmission and reception. For each game, 370 T2^∗^-weighted whole-brain echo-planar images depicting the blood oxygen level-dependent (BOLD) contrast were acquired with the following parameters: slice thickness = 2.4 mm; inter-slice gap = 1 mm; TR = 2000 ms; TE = 25 ms; flip angle = 75°; in-plane voxel dimension = 3.4 mm; number of slices = 38; dummy acquisitions = 4 and matrix = 64 × 64. For anatomical reference, a whole-brain high-resolution gradient-echo image of 43 slices was acquired with the following parameters: slice thickness = 3 mm; inter-slice gap = 0.3 mm; TR = 3000 ms; TE = 30 ms; flip angle = 90°; in-plane voxel size = 1.9 mm and matrix = 128 × 128. Participants were placed head first in the scanner. Foam padding was placed around the head in the coil to minimize head movement and the participants were provided with ear protectors. The participants looked at the screen through a mirror. Participants were equipped with a button box in their right hand. One button was used to increase the investment, one to decrease the investment.

Data were analyzed with SPM12^1^. All images were corrected for head-motion using iterative rigid body realignment with six motion-parameters to minimize the residual sum of squares between the images. The functional images of each subject were co-registered to that subject’s structural scan. The functional images were spatially normalized (“old normalized”) using the Montreal Neurological Institute (MNI) 152 T1 template (voxel size = 3.5 × 3.5 × 3.5), and spatially smoothed using an 8-mm full-width, half-maximum Gaussian kernel, to allow for group-analyses. Per subject 370 scans were acquired per condition.

At first-level, fMRI time-series data were modeled by a series of events convolved with a canonical hemodynamic response function (HRF). The investment phase was modeled as an event lasting from the start of the investment phase until the moment the participant pressed the button to make the investment, or to choose the indicated number in the control condition (mean reaction time 3.7 s, *SD* = 0.93 s). The repayment phase was the period during which the response of the trustee was shown, lasting 3 s (see Figure 1). Game trials were contrasted with the corresponding phases of the control trials. Six movement parameters were included in the model.

Analogous to our previous study (Lemmers-Jansen et al., 2017), ROI analyses were conducted on the right TPJ (MNI coordinates: 45, -43, 32), right dlPFC (51, 18, 30), right insula (36, 24, 0) and the ACC (-3, 27, 33), complemented with the left TPJ (-44, -46, 29), ventral striatum (VS; 14, 12, -5), amPFC (0, 42, 6), and bilateral caudate ROI’s (right: 6, 11, 5; left: -7, 12, -4). ROIs were defined as a 10 mm sphere around the given coordinates, except for the caudate, where a 5 mm sphere was used. Analyses were conducted in SPM12, using Marsbar-0.44^2^ to generate the ROIs. We used an event related, factorial design with gender as contrast and age as covariate. All ROI analyses were conducted separately for the investment and repayment phase, in the cooperative and unfair conditions.

Additionally, exploratory whole-brain analyses were performed to examine group wise differences in regions outside the a priori defined ROIs. The results are presented in the Supplementary Material (Supplementary Tables S2–S4).

## Results

### Participant Characteristics

Participant characteristics are described in Table 1. There were no group differences between males and females in age. However, WASI vocabulary scores differed significantly between males and females, with males scoring on average 6 points higher than females. There was no significant correlation between WASI scores and investment, suggesting that any gender differences in investment were unlikely influenced by systematic differences in general cognitive ability. One third of the participants indicated doubts in response to the question if they believed they were interacting with a real partner. However, no differences in investments and ROI activation were found between the participants with and without doubts (*p* > 0.7 and *p* > 0.4, respectively), and analyses without those who had doubts that the trustee was real yielded similar outcomes as the results presented below. Several strategies were used during investments (see Table 1), but these did not differ significantly between genders.

### Behavioral Results

**FIGURE 2:**
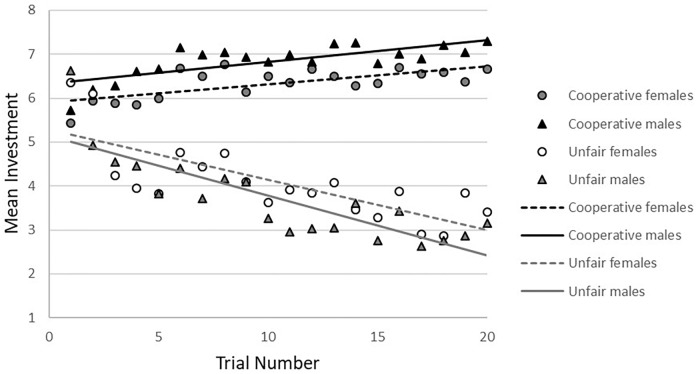
Mean investment over trials by gender and condition of the trust game.

The investments in the trust game are shown in Table 1. The effect of condition on investment was investigated as a manipulation check. Results showed significant differences between conditions (see Figure 2), indicating that the task conditions (cooperative vs. unfair) worked as intended (*b* = 2.72, *p* < 0.001, 95%CI = -2.89/-2.55).

For baseline trust there were no gender-by-age interaction (β = -0.23, *p* = 0.83) or significant main effects of gender or age (β = 0.13, *p* = 0.22 and β = 0.08, *p* = 0.42, respectively).

**FIGURE 3:**
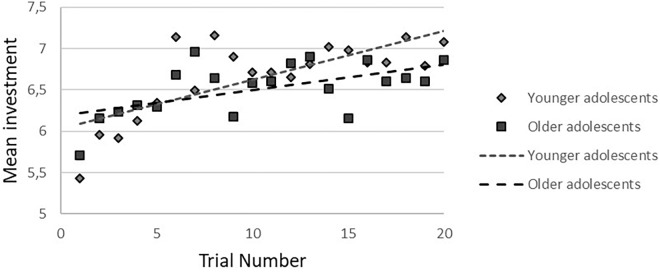
Age-by-trial number interaction in younger and older adolescents during cooperation. To visualize the effect, a median split for age was performed.

In the *cooperative condition*, no gender-by-age-by trial number interaction was found. After the three-way interaction was removed from the model, a gender-by-age interaction at trend level (*b* = -0.40, *p* = 0.09, 95%CI = -0.85/0.06) was observed. There was a significant age-by-trial number interaction (*b* = -0.11, *p* = 0.032, 95%CI = -0.02/-0.001), showing that younger participants increased their investments more than older participants (younger: *b* = 0.05, *p* < 0.001, 95%CI = 0.32/0.08; older: *b* = 0.04, *p* < 0.001, 95%CI = 0.01/0.06), based on a median age split (age 16.9; see Figure 3).

**FIGURE 4:**
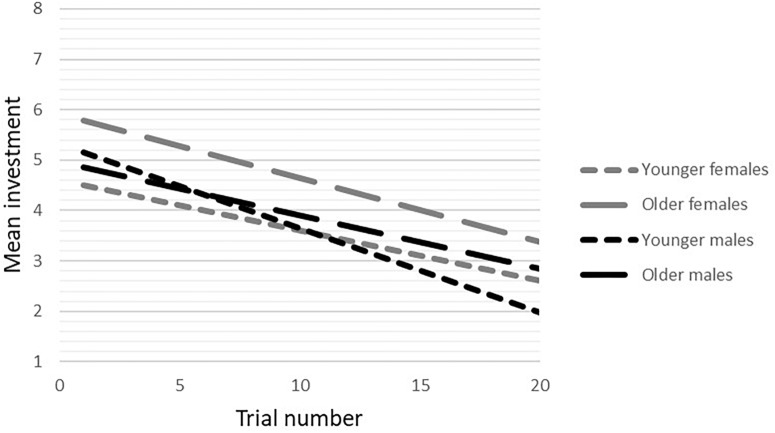
Gender-by-age interaction on investments over trials in the unfair condition.

In the *unfair condition*, there was a significant gender-by-age-by-trial number interaction (*b* = 0.03, *p* < 0.03, 95%CI = 0.003/0.05). Analyses by gender showed a significant interaction between age and trial number on investment in males (*b* = 0.02, *p* < 0.05, 95%CI = 0.001/0.04), but not females (*b* = -0.01, *p* = 0.32, 95%CI = -0.03/0.01). *Post hoc* analyses with a median split for age showed that younger males decreased their investments more strongly toward the unfair other than older males (see Figure 4). In females there was a significant main effect of age (*b* = 0.34 *p* < 0.05, 95%CI = 0.02/0.66), showing that younger females invested less in the unfair partner than older females (see Figure 4), but there was no significant main effect of trial number.

### fMRI ROI Results

#### Cooperative Interactions

**FIGURE 5:**
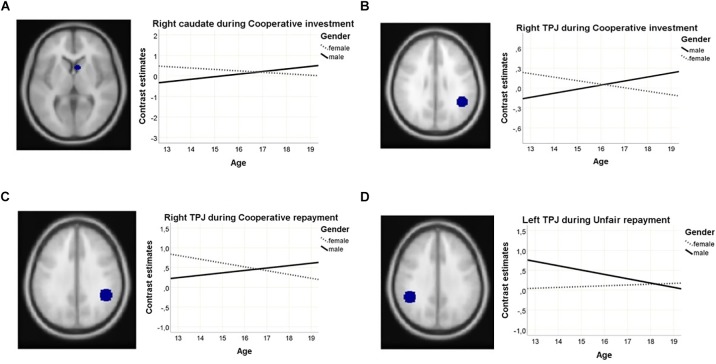
Gender-by-age interactions in ROI activation showing **(A)** the right caudate during cooperative investments; **(B)** the left TPJ during cooperative investments; **(C)** the right TPJ during cooperative repayments; and **(D)** the left TPJ during unfair repayments.

ROI analysis revealed gender-by-age interactions in the cooperative investment phase, in the left TPJ and the right caudate (see Figure 5). During the cooperative repayment phase, a gender-by-age interaction was found with a significance level just bordering the threshold adjusted for multiple comparisons in the right TPJ (see Figure 5). All areas showed greater increase of activation with age in males compared to females. Main effects of gender, bordering significance, became apparent in the cooperative repayment phase (see Table 2), with males activating the TPJ more, and females activating the caudate more. There was no main effect of age.

**Table 2:** ROI analyses outcome, by condition of the trust game.

Condition Association	ROI	*p*	*t*
**Cooperative investment^∗^**			
Interaction age and gender:			
Age males > age females	Left TPJ	0.019	2.14
	Right caudate	0.015	2.26
**Cooperative repayment^∗∗^**			
Interaction age and gender:			
Age males > age females	Right TPJ	0.037^#^	1.84
**Main effect of gender:**			
Males > females	Left TPJ	0.036^#^	1.85
Females > males	Left caudate	0.034^#^	1.87
**Unfair investment^∗∗∗^**			
Increasing with age	ACC	0.003	3.13
	dlPFC	0.04^#^	1.79
**Unfair repayment^∗∗^**			
Interaction age and gender: Age females > age males	Left TPJ	0.031	1.93


*Note: All ROIs were defined as a 10 mm sphere (except right caudate: 5 mm) around the following MNI coordinates: right temporo-parietal junction (TPJ): 45, -43, 32; left temporo-parietal junction: -44, -46, 29; right caudate: 6, 11, 5; left caudate -7, 12, -4; dorso-lateral prefrontal cortex (dlPFC): 51, 18, 30; anterior cingulate cortex (ACC): -3, 27, 33. ^∗^adjusted threshold for the cooperative investment phase: *p* = 0.037. ^∗∗^adjusted threshold for the cooperative and unfair repayment phase: *p* = 0.032. ^∗∗∗^adjusted threshold for the unfair investment phase: *p* = 0.036. ^#^bordering significance of the adjusted *p*-value.*

#### Unfair Interactions

During the repayment phase, a gender-by-age interaction was found in the left TPJ, with greater increase of activation with age in females compared to males (see Figure 5). There were no significant main effects of gender. In the ACC and dlPFC, a non-significant trend-level effect of age was found, showing increased activation in older participants during investments.

## Discussion

This study set out to investigate the development of trust in adolescent boys and girls. Using two multi-round trust games, we found gender-by-age interactions on investment behavior during unfair interactions, with younger males reacting more strongly to unfair partner feedback. During cooperative interactions there was a significant age-by-trial number interaction, showing that younger participants increased their investments slightly more than older participants. At the neural level, significant gender-by-age interactions and main effects of gender bordering significance were found in the TPJ and caudate, suggesting differential cognitive mechanisms underlying trust between genders that change during this phase of development. Age-related increases of activation in cognitive control areas were found at trend level and only in unfair interactions.

### Behavioral Findings

#### Baseline Trust

Contrary to our hypothesis and previous results, baseline trust did not differ significantly between genders in this adolescent sample. Adult males tend to trust more than females (Sutter and Kocher, 2007; Buchan et al., 2008; Croson and Gneezy, 2009; Van den Akker, 2018). This pattern was also found in our older adolescent sample (Lemmers-Jansen et al., 2017), and in a mid-adolescence sample (14–16.5 years) using a repeated one-shot trust game (Derks et al., 2014). These findings are contradictory, especially with Derks et al. (2014). This could be due to differences in the experimental set-up and needs to be investigated further.

No age-related changes in baseline trust were found, suggesting that baseline trust does not increase substantially from early to late adolescence. Possibly, age-related changes in baseline trust during adolescence are small, with variability throughout this phase of development, and thus are only detectable when looking at a larger time window [see also van den Bos et al. (2010, 2011, 2012); Fett et al. (2014a)].

#### Repeated Interactions

Changes in trust in response to cooperative feedback only showed a trend-level gender-by-age interaction, and no main effects of gender. Younger adolescents, however, showed a steeper increase of investments than older adolescents. The finding of absent gender effects during adolescence are in line with our previous study in slightly older adolescents and young adults (Lemmers-Jansen et al., 2017). Gender differences in repeated trust games have rarely been investigated. In males it has been found that investments increase with age from adolescence to mid adulthood (Fett et al., 2014a), thus it might be likely that gender differences also emerge later during development when gender roles become more established or specific cognitive abilities more refined. The current results do not support earlier work by van den Bos et al. (2010, 2012), who found age-related increases in reciprocity during development (age 9–25, and mean age 11, 16, and 19, respectively), using a two-choice trust game. It is possible that the development of trust and reciprocity follows different developmental trajectories.

During unfair interactions a gender-by-age-by-trial number interaction on levels of trust was found. The direction of the interaction, however, did not correspond with our hypothesis. All age groups adjusted levels of trust in response to unfair feedback, reflected in lower investments over time. Overall, younger individuals showed lower trust. Contrary to our expectation, younger males showed a steeper decline of investment than older males. This result contradicts our previous findings, where similar behavior was found in older, and not in younger males (Lemmers-Jansen et al., 2017). Younger females made lower investments than older females.

The current results suggest that the response to cooperation in females develops in a linear way, and that the development of trust in males might level off towards adulthood. However, responses to breaches of trust in males showed a different development, with a less drastic response to unfair partner behavior during later adolescence. Females seem to follow a more linear pathway in the development of trust in response to unfair feedback, with slightly higher investments in older females. Lower investments might reflect greater weariness of the unfair partner or less attempts to establish cooperation.

### Neural Results

In contrast to the behavioral findings in the cooperative condition that showed similar levels of trust in males and females, at the neural level several gender-by-age interactions were found in the TPJ and caudate. These areas have been consistently found in the trust game, and have been linked to the mentalizing and reward learning components of the trust game, respectively (King-Casas et al., 2005; Lee, 2008; van den Bos et al., 2011; Lemmers-Jansen et al., 2017). In the investment phase, the activation in females in the left TPJ and right caudate decreased with age, whereas in males activation increased with age. The same pattern, however, at trend level, was found in the right TPJ during repayments. In the trust game and other social cognitive tasks, gender differences in TPJ activation have been reported in young adults, with higher activation in males compared to females (Schulte-Rüther et al., 2008; Luo et al., 2015; Lemmers-Jansen et al., 2017), but the reverse has also been reported (Chan, 2016; Zhang et al., 2017). Gender differences in activation of the caudate in response to emotional stimuli have been reported, showing higher activation in females [for a meta-analysis, see Stevens and Hamann (2012)]. In absence of behavioral differences, these results could suggest that males and females have different motivations or strategies for the same behavior, or adopt different cognitive strategies in response to processing social feedback (Cahill, 2006). These gender differences in strategies or motivations change with age. Apart from different strategies and motivations, these gender-by-age interactions may also point toward gender differentiated development in the given areas, which were not observed in the older sample (Lemmers-Jansen et al., 2017).

During unfair interactions, a gender-by-age interaction was observed in the right TPJ, with females showing slightly increasing activation with age, and males showing reduced activation with increasing age. In combination with the behavioral findings, this suggests that younger males respond stronger to negative feedback than older males, indicating increased efforts to mentalize about the other’s behavior and a stronger tendency to retaliate untrustworthy behavior.

Only under unfair treatment by the other player, age-related changes in neural activity became apparent, in the ACC and at trend level in the dlPFC. The age-related changes in the ACC during unfair interactions are in line with findings in an overlapping sample (Fett et al., 2014a), which included a much larger age range. Increasing activation with age in ACC and dlPFC have been also reported by van den Bos et al. (2011), however, in decisions to trust compared to no trust decisions. These regions are associated with conflict processing and the cognitive control network (MacDonald et al., 2000; Botvinick et al., 2004; Pochon et al., 2008), which is still developing during adolescence (Fair et al., 2007; Kelly et al., 2009; Crone and Dahl, 2012; Steinbeis et al., 2014; Crone and Steinbeis, 2017). With increasing age, increasing activation of the dlPFC was found in the unfair condition, suggesting that cognitive control areas are more engaged during decisions to (dis)trust across adolescent development. The current findings are also in line with Van Duijvenvoorde et al. (2008) and Van Den Bos et al. (2009). Using non-social rule selection and probabilistic learning tasks, based on learning from positive and negative feedback in a similar developmental sample, they found that both ACC and dlPFC were activated more with increasing age during negative feedback processing.

### Limitations and Future Directions

The current findings need to be interpreted in the light of several limitations. Firstly, developmental changes in trust seem to be subtle. In order to investigate gender specific development, a larger age-range might have to be included as changes may be most obvious during transition to adulthood (Fett et al., 2014a). Secondly, participants played against a computerized algorithm, rather than a human counterpart. One third of the participants said that they doubted that the other person was real. Analyses comparing the behavior of the individuals who expressed or did not express doubts did not yield significant differences in terms of investments. In addition, higher investments in the cooperative condition and lower investments in the unfair condition showed that overall the experimental manipulation of the counterpart was effective. Moreover, we informed participants that they were paid upon performance in the trust game, aiming to increase task engagement. Additionally, despite the advantages of experimental investigations, they face the problem of generalizability to other, real world settings. The findings therefore should be considered with caution, when making generalizations to other contexts.

Different motives may underlie trust game behavior. For example, a reduced adjustment to unfair behavior of the partner may be associated with perspective-taking ability (Fett et al., 2014b), but also with an inclination to restore trust. Future studies may shed further light on underlying motives by including detailed experimental and questionnaire measures such as social value orientation (Derks et al., 2014, 2015), and Machiavellianism (Bora et al., 2009; Čavojová et al., 2011), or by specific experimental manipulations of the game. Underlying mechanisms and motivations may be revealed with fMRI (i.e., through activation of particular areas that have typically been associated with particular functioning by other studies), however, these interpretations rely on reverse inference (Poldrack, 2006; Poldrack et al., 2016). No firm conclusions can be drawn from these data, but they provide a starting point for generating new hypotheses. These hypotheses in turn warrant further investigation and testing in future research.

In summary, we set out to investigate the neural mechanisms underlying gender and age effects on social interactions using a trust game during functional MRI. Results showed that there were no gender and age differences in baseline trust, and age differences in the increase of investments over trials during cooperative interactions, with younger adolescents showing a slightly steeper increase over repeated interaction. The findings suggest relatively stable processes of trust and cooperation between 13 and 19 years of age. During unfair interactions, younger males showed stronger sensitivity to unfairness, suggested by a stronger increase in distrust than older males. In females, age was associated with higher overall investments. The current study suggests that younger adolescents are more sensitive to their partner’s trustworthiness. Differential patterns of neural activation may suggest different cognitive strategies underlying similar behavior in males and females. Specifically, mentalizing and reward-related areas were differentially activated in males and females, and also showed different age-specific trends. Future studies need to investigate these mechanisms further.

## Ethics Statement

This study was approved by the local research ethics committee [London-Surrey Borders (10/H0806/38)].

## Author Contributions

LK and A-KF designed the experiments. SS and LK supervised the study. A-KF collected the data. MvK and IL-J analyzed the data. IL-J, A-KF, MvK, SS, and LK interpreted the results. IL-J wrote the manuscript. All the authors discussed the results, reviewed and contributed to the critical development of the manuscript, and approved the final manuscript.

## Conflict of Interest Statement

The authors declare that the research was conducted in the absence of any commercial or financial relationships that could be construed as a potential conflict of interest.

## Footnotes

1 http://www.fil.ion.ucl.ac.uk/spm/

2 http://marsbar.sourceforge.net/
