# Supplementary material for: Girls-Boys: An Investigation of Gender Differences in the Behavioral and Neural Mechanisms of Trust and Reciprocity in Adolescence
Source: Front Hum Neurosci. 2019 Aug 2;13:257. doi: 10.3389/fnhum.2019.00257 (PMC6688065; doi:10.3389/fnhum.2019.00257)
Supplement: Supplementary file 1 [file Data_Sheet_1.pdf]

Supplementary Material

Girls-Boys:

An Investigation of Gender Differences in the Behavioral and Neural Mechanisms of Trust and Reciprocity in Adolescence

Lemmers-Jansen et al.

Supplementary Material

Table S1
Participant Characteristics of the fMRI Sample

	Male<br>*N* = 24	Female<br>*N* = 20	Statistics		Overall<br>*N* = 44
**Measures**	Mean (*SD*)	Mean (*SD*)	*Beta*	*p*	Mean (*SD*)
Age	16.84 (1.63)	16.9 (1.47)	.03	.83	16.85 (1.54)
WASI scaled	65.38 (9.97)	48.2 (9.41)*	.4	.008	52.67 (10.66)
	Division per group, *N (%)*		χ^2	*p*	Division per group, *N (%)*
Education level #			37.3	<.001	
- 2	1 (4%)	8(47%)			9 (22%)
- 3	-	9 (53%)			9 (22%)
- 4	23 (96%)	-			23 (56%)
**Measures**	Mean (*SD*)	Mean (*SD*)	*Beta*	*p*	Mean (*SD*)
First investment, baseline trust	6.0 (2.3)	5.5 (2.7)	.11	.47	5.8 (2.5)
Mean investment Cooperative partner	6.7 (2.8)	6.5 (2.7)			6.6 (2.8)
Mean investment Unfair partner	4.0 (3.3)	4.3 (3.1)			4.1 (3.2)
	N (%)	*N* (%)	χ^2	*p*	*N* (%)
After trust game questionnaire #					
Manipulation doubt?	9 (39%)	3 (23%)	.63	.43	12 (33%)
Strategy:			1.22	.88	
- responding to partner	7 (32%)	5 (33%)			12 (32%)
- maximize profit	5 (23%)	3 (20%)			8 (22%)
- no strategy	8 (36%)	4 (27%)			12 (32%)
- other	2 (9%)	3 (20%)			5 (14%)

** = Significant difference at p < .01*
# = Missing data of 3 females for education level;1 male and 5 females for the manipulation check; and 2 males and 5 females for the strategy questionnaire.
Note: WASI vocabulary = Wechsler Abbreviated Scale of Intelligence, vocabulary subscale.
After the trust game participants were asked if at any time they had doubts whether their counterpart was real. If they responded personalizing (saying 'he'), it was coded as believing the counterpart was real. If in two conditions they reported probabilistic answers, predictable or unreal, then it was coded as having doubts. Then participants were asked about the strategy used for investment. If the answer included the behaviour of the counterpart, it was coded as 'responding to partner'; if the answer contained 'great', 'maximum', 'profit', or 'more than the other' it was coded as 'maximize profit'; some participants answered the did not use a strategy; and other comprises 'random', 'gambling', or always trying the same amount.

Whole-brain additional analysis

A one-sample t-test with age as covariate of interest was used to investigate the effects of age and a two-sample t-test was used for the effects of gender on brain activation during the four

conditions of the trust game. Further, a 2-sample t-test (male vs female) with age as a covariate was used, to investigate the differential effect of age on brain activation between genders. For exploratory purposes, results are shown here at a more lenient threshold of p = .001 uncorrected. All clusters equal to and larger than 10 voxels are reported and p-values are FWE-cluster corrected.

Table S2

Age-by-Gender Interactions in Whole-Brain Activation by Trust Game Condition

Condition	Region	Cluster size	MNI coordinates X	Y	Z	Hemisphere	*Z*	*p*
Cooperative investment, males with age > females								
	Middle frontal gyrus	85	-30	44	10	L	3.46	0.54
	TPJ	21	-54	-44	34	L	3.31	0.83
Cooperative repayment, males with age > females								
	vlPFC	23	-38	40	0	L	3.41	0.86
	vlPFC	13	48	8	16	R	3.36	0.91
	vlPFC	12	-50	8	12	L	3.44	0.91
Unfair investment, males with age > females								
	Precentral gyrus	41	-34	-6	34	L	3.44	0.74
Unfair repayment, females with age > males								
	Orbitofrontal gyrus	140	58	-4	18	R	3.88	0.31
	Middle frontal gyrus	19	38	42	32	R	3.75	0.89
	Superior frontal gyrus	53	18	-10	74	R	3.65	0.69
	SMA		10	-4	70		3.17	
	Middle frontal gyrus	41	-40	36	32	L	3.52	0.76
	Mid cingulum	108	8	-20	42	R	3.45	0.42
			6	-6	42		3.17	
	Mid cingulum	34	-8	-26	40	L	3.28	0.80
	Mid cingulum	12	-8	-2	40	L	3.21	0.92
	Postcentral gyrus	613	-46	-32	54	L	4.06	0.007
	Inferior parietal gyrus		-42	-46	60		4.05	
	Superior parietal gyrus		-32	-48	66		3.90	
	Superior temporal gyrus	15	50	-10	6	R	3.28	0.91
	TPJ	14	-50	-52	20	L	3.19	0.91
	Supra marginal gyrus	15	-54	-22	22	L	3.22	0.91
	Cerebellum	11	-30	-64	-30	L	3.26	0.93
	Cerebellum	13	-2	-72	-22	L	3.20	0.92

Note: Results are shown at a lenient threshold of p = .001 uncorrected. All clusters with a minimum size of k = 10 are reported and p-values are Family Wise Error-cluster corrected. TPJ = temporo-parietal junction; vlPFC = ventrolateral prefrontal cortex; SMA = supplementary motor area.

Table S3

Gender Differences in Whole-Brain Activation by Trust Game Condition

Condition	Region	Cluster size	MNI coordinates X	Y	Z	Hemisphere	*Z*	*p*
Cooperative investment, males > females								
	vlPFC	29	58	12	16	R	3.48	0.79
Cooperative repayment, females > males								
	Precentral gyrus	31	-36	-12	64	L	3.39	0.81
	Superior temporal pole	220	28	10	-18	R	4.70	0.17
			35	12	-24		4.06	
	Superior parietal gyrus	21	34	-50	66	R	3.33	0.87
	Insula	166	-34	12	4	L	3.83	0.27
			-38	8	-10		3.53	
	Amygdala	25	-22	4	-18	L	3.70	0.84
Unfair repayment, males > females								
	vlPFC	16	-48	14	14	L	3.45	0.90
	Mid cingulum	38	-16	-36	36	L	3.41	0.77
	Insula	92	-36	-2	4	L	3.56	0.49
	Middle temporal gyrus	11	-62	-20	-6	L	3.38	0.92
	TPJ	21	-60	-40	26	L	3.30	0.87
	Putamen		-32	-14	8		3.38	
	Putamen	22	30	-6	14	R	3.26	0.86

Note: Results are shown at a lenient threshold of $p = .001$ uncorrected. All clusters with a minimum size of k = 10 are reported and *p*-values are Family Wise Error-cluster corrected. vlPFC = ventrolateral prefrontal cortex; TPJ = temporo-parietal junction

Table S4

Association Between Age and Whole-Brain Activation by Trust Game Condition

Condition	Region	Cluster size	MNI coordinates X	Y	Z	Hemisphere	*Z*	*p*
Cooperative investment, decreasing with age								
	Mid cingulum	21	8	0	28	R	3.65	0.83
	Mid cingulum	24	-8	-4	30	L	3.78	0.82
	Middle temporal gyrus	12	-50	2	-22	L	3.21	0.88
Cooperative repayment, decreasing with age								
	Inferior occipital gyrus	12	30	-96	-10	R	3.69	0.91
Unfair investment, increasing with age								
	Middle frontal gyrus	81	-30	44	24	L	3.53	0.55
			-40	45	22		3.22	
	Middle frontal gyrus	40	-28	28	30	L	3.29	0.74
	Superior temporal pole	286	-48	8	-10	L	3.95	0.12
	Insula		-38	10	-10		3.87	
			-30	18	-8		3.42	
	Precentral gyrus	125	-44	2	22	L	3.53	0.39
	vlPFC		-45	8	10		3.31	
	ACC	376	0	10	30	R	4.27	0.06
	Mid cingulum		-8	18	38	L	3.10	
	Mid cingulum	29	-2	10	44	L	3.30	0.80
	Middle occipital gyrus	27	-26	-92	6	L	3.46	0.81
	Superior occipital gyrus	17	-14	-96	14	L	3.41	0.86
	Inferior occipital gyrus	53	-38	-76	-10	L	3.32	0.68
	Inferior temporal gyrus		-46	-55	-12		3.28	
	Lingual gyrus	268	12	-82	-8	R	3.90	0.13
	Calcarine		-8	-88	-5	L	3.40	
	Lingual gyrus	56	-24	-88	-14	L	3.63	0.66
Unfair repayment, increasing with age								
	Superior temporal pole	40	-36	8	-24	L	4.16	0.76
Unfair repayment, decreasing with age								
	ACC	101	-2	50	2	L	3.52	0.46
	Orbitofrontal gyrus		0	40	-6	R	3.44	
	Precentral gyrus	62	-44	-6	48	L	3.97	0.64
	Postcentral gyrus	70	-24	-48	58	L	3.53	0.60
	Postcentral gyrus	19	22	-46	56	R	3.33	0.88
	Postcentral gyrus	12	-32	-30	44	L	3.24	0.92
	Middle temporal gyrus	147	46	-46	16	R	4.07	0.30
	Middle temporal gyrus	330	-50	-58	6	L	4.00	0.06

Middle temporal gyrus	57	-56	-40	12	L	3.61	0.67
Superior temporal gyrus	83	46	-20	-6	R	3.54	0.53
Superior parietal gyrus	68	-22	-72	58	L	3.41	0.61
		-22	-66	52	L	3.37	
Precuneus	26	-12	-56	24	L	3.37	0.84
Cerebellum	15	14	-56	-42	R	3.32	0.90

Note: Results are shown at a lenient threshold of p = .001 uncorrected. All clusters with a minimum size of k = 10 are reported and p-values are Family Wise Error-cluster corrected. ACC = anterior cingulate cortex; vlPFC = ventrolateral prefrontal cortex;
